# Coding accuracy on the psychophysical scale

**DOI:** 10.1038/srep23810

**Published:** 2016-03-29

**Authors:** Lubomir Kostal, Petr Lansky

**Affiliations:** 1 Institute of Physiology, Academy of Sciences of the Czech Republic, Videnska 1083, 14220 Prague 4, Czech Republic

## Abstract

Sensory neurons are often reported to adjust their coding accuracy to the stimulus statistics. The observed match is not always perfect and the maximal accuracy does not align with the most frequent stimuli. As an alternative to a physiological explanation we show that the match critically depends on the chosen stimulus measurement scale. More generally, we argue that if we measure the stimulus intensity on the scale which is proportional to the perception intensity, an improved adjustment in the coding accuracy is revealed. The unique feature of stimulus units based on the psychophysical scale is that the coding accuracy can be meaningfully compared for different stimuli intensities, unlike in the standard case of a metric scale.

The efficient coding hypothesis^1^ states that neuronal responses are adjusted, through evolutionary and adaptive processes, to optimally encode such stimulus statistics that matches the sensory environment^2^,^3^,^4^. The statistics of many natural stimuli differs, over short timescales, from the average global distribution, and typically, the limited neural coding range does not cover the wide range of possible stimuli values^5^,^6^,^7^. The efficient coding hypothesis therefore predicts that neurons adapt their coding properties to the local stimulus distribution^8^. In particular, the coding accuracy should increase near the most commonly occurring stimuli in order to minimize the overall decoding error and to maintain the efficient representation of the environment. Such situation is reported in the auditory coding of the sound intensity^5^,^6^,^9^,^10^, of the interaural level differences^11^ and time differences^12^, but also in the neural coding in the primary visual cortex^7^ and primary somatosensory cortex^13^. The coding accuracy is commonly evaluated by means of the stimulus-reconstruction paradigm^14^, that is, by answering how well may the ideal observer determine the stimulus value from the noisy neuronal response. It is assumed that the inverse of the Fisher information approximates the minimal mean squared error^9^,^15^,^16^,^17^,^18^,^19^,^20^,^21^. Higher Fisher information reflects higher coding accuracy so that a more precise representation of stimuli is possible.

The goal of this short paper is to point out to a potentially problematic aspect of aligning the maximal coding accuracy with the most frequent stimuli. Our reasoning follows from the fact that the stimulus values are quantified by choosing some convenient, but otherwise *arbitrary*, system of measurement units. For example, the sound intensity is typically expressed as the sound pressure level in decibels (dB SPL). The same stimulus intensity can be equivalently expressed in terms of the effective pressure in Pascals (Pa)^22^. The seemingly arbitrary choice of stimulus scale, however, has a non-trivial and significant impact on the coding precision. As demonstrated by Kostal and Lansky^23^, a non-linear relationship between different units (such as between the sound pressure and the sound level) may affect the position of maximal coding accuracy. Here we demonstrate the paradoxical consequences of the stimulus scale change on *both* the coding accuracy and the known stimulus distribution simultaneously. We show that the match between high coding accuracy regions and most frequent stimuli regions depends on the choice of the measurement unit. Second, we attempt to resolve this problem by arguing that the natural system for stimulus quantification is given by the scale linearly proportional to the perception intensity^24^,^25^. As an illustration, we employ the classical Riesz’s psychophysical scale for the sound intensity^26^ to reveal the expected coding accuracy adaptation even for low pressure levels in the experimental data of Watkins and Barbour^9^.

## Methods

The psychophysical scale describes the perceptual intensity, *ψ*, as a function of the stimulus intensity *I*^24^,^25^. The empirical finding known as Weber’s law^27^,^28^ states that the smallest noticeable increment in perception, Δ*ψ*, remains constant if the *relative* stimulus increment (also known as Weber’s factor) is also constant,


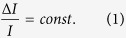


As suggested later by Fechner^29^, Weber’s law effectively sets the scale for the perceived stimulus intensity since Δ*I*/*I* is proportional to Δ*ψ*. By integrating Eq. (1) we obtain the well known Fechner’s law, stating that the perceived intensity varies as^28^.


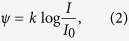


where *k* is a proportionality factor and *I*_0_ some reference value.

Subsequent investigations found that Eq. (1) holds neither generally nor exactly^24^,^30^ across different sensory modalities. In particular, Weber’s factor for human sound intensity discrimination was found to satisfy^26^,


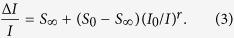


Here *I* is the basal sound intensity in W/m^2^, Δ*I* is the minimum perceptible difference, *S*_∞_ is the value Δ*I*/*I* approaches at high intensities, *S*_0_ > *S*_∞_ is the value of Δ*I*/*I* at the threshold of hearing and *r* is a parameter, approximately *r* = 1/2. Weber’s factor in Eq. (3) is no longer constant, but decreases rapidly to a plateau with increasing intensity *I*. Since the sound intensity and the sound pressure are related by the acoustic impedance 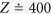 N.s.m^−3^ as^22^


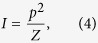


the following differential equation follows from Eq. (3)


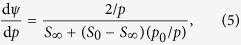


provided that the derivative d*ψ*/d*p* is a good approximation to Δ*ψ*/Δ*p*. The solution to Eq. (5) is


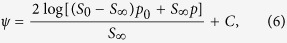


which is proportional to log(*c* + *p*). Setting the values^26^ *S*_∞_ = 0.2, *S*_0_ = 1 and arbitrary *p*_0_ = 20 *μ*Pa (the actual reference level is of little importance here) we obtain


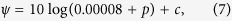


where 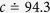 so that *p* = 0 Pa yields *ψ* = 0 for convenience. Eqution (7) determines Riesz’s scale (in arbitrary units) of sound pressure values, correcting the inadequate Fechner’s law in Eq. (2) for small sound intensities (pressures). In other words, the value of *ψ* can be used to measure the sound intensity on the scale which is linearly related to the perception intensity. The standard sound pressure level scale *L* (given in dB SPL) is essentially equivalent to Fechner’s law, since due to Eq. (4) it holds^22^


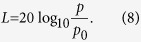


**Figure 1:**
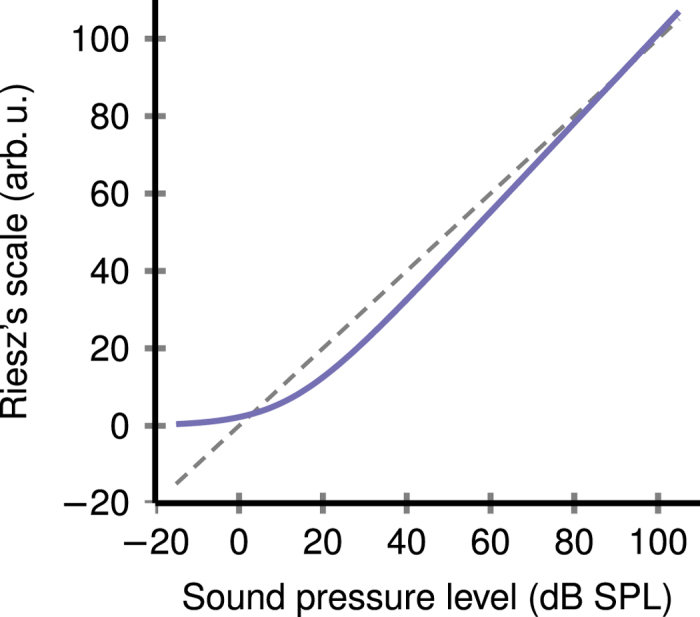
Relationship between two possible measurement scales for the sound intensity. The psychophysical scale by Riesz as a function of the sound pressure level (solid) is significantly non-linear only for low sound intensities. The identity function is shown for comparison (dashed).

The Eqs (7) and (8) are approximately proportional to each other for sufficiently high pressure levels (Fig. 1).

## Results

The coding accuracy as a function of the stimulus intensity is significantly affected by the choice of the measurement scale^23^. The question is whether the coding accuracy adaptation to the stimulus distribution (as observed, e.g., in the experiments^5^,^6^,^9^), is preserved under the change of stimulus units.

The Fisher information *I*_F_(P) as a function of the sound pressure, and the Fisher information *I*_F_(ψ) for the sound intensity measured on Riesz’s scale from Eq. (7), are related as


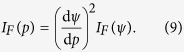


Similarly, one may additionally use Eq. (8) to relate, e.g., *I*_*F*_(*ψ*) and *I*_*F*_(L). The transformation rule in Eq. (9) is well known and can be derived directly from the definition of the Fisher information by using the chain rule for derivatives^15^. Similarly, the stimulus probability density function *f*(·) satisfies^31^


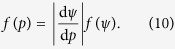


Therefore it follows that any visual alignment between the values of the coding accuracy and the stimulus distribution depends crucially on the choice of units. Even though the square root of the Fisher information transforms analogously to Eq. (10), the potential match between the peaks of 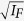 and *f* is *not preserved* under the stimulus scale change because 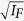 and *f* are often related non-linearly. In fact, it can be shown rigorously that also the global match between the profiles of 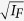 and *f* is *not preserved* under the stimulus scale change^32^, unless the stimulus probability density function is *exactly* proportional to the square root of the Fisher information (known as the Jeffreys prior^33^).

We illustrate how a specific choice of the stimulus units improves the experimentally observed adaptation of the coding accuracy to the stimulus distribution. We argue that the stimulus scale proportional to the actual perception intensity (the psychophysical scale) is the natural reference frame under which the coding accuracy should be evaluated.

**Figure 2:**
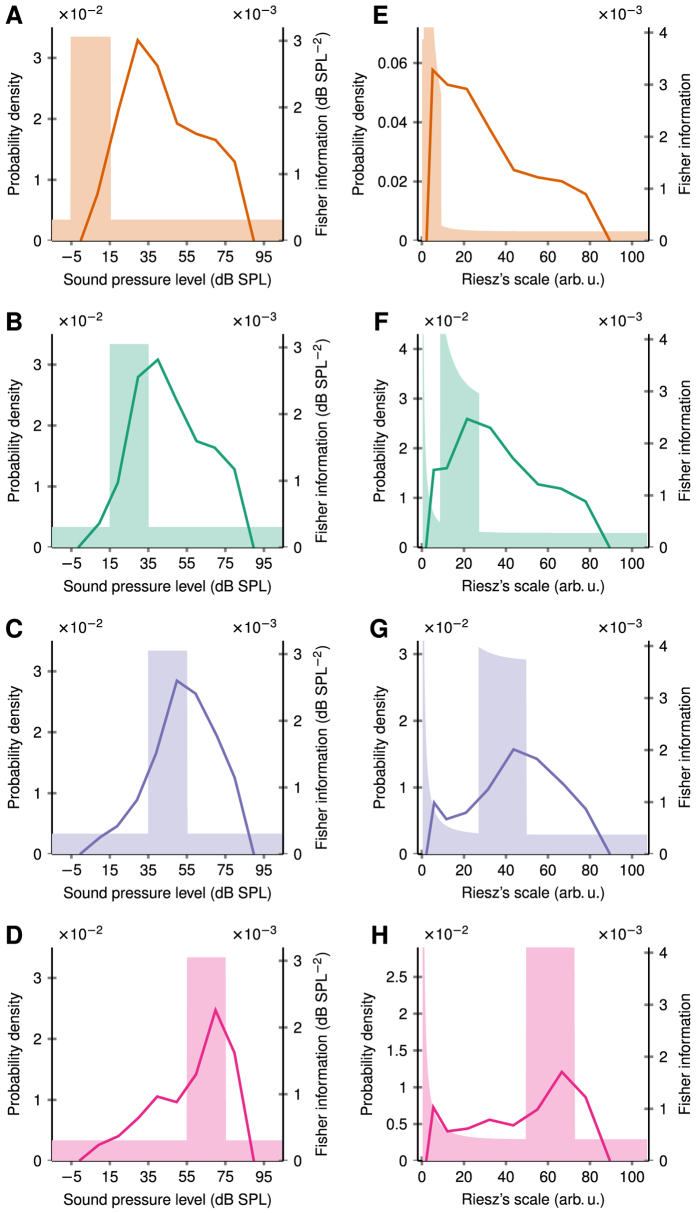
Maximal coding accuracy aligns with stimulus statistics only on the proper stimulus measurement scale. Colored area indicates the stimulus probability density function, solid line is the coding accuracy (Fisher information). (**A–D**) Original data reconstructed from Watkins and Barbour^9^ show weak adaptation of the coding accuracy to frequent low-intensity sounds (**A**) on the sound level scale (dB SPL). The alignment improves as the high-probability stimulus region moves towards higher sound intensities (**B,C**). (**E–H**) The same data plotted on Riesz’s scale of sound intensities (in arbitrary units) reveal that the coding accuracy is actually perfectly adjusted for all four stimulus distributions.

Neurons in the auditory system are reported to adjust their rate-intensity functions in order to improve coding accuracy over high-probability stimulus regions^5^,^9^,^10^. The match is not perfect for low sound intensities and a positive bias of maximal coding accuracy towards higher intensities is reported. For example, in the experiment of Watkins and Barbour^9^, the sound level distribution was set to be uniform over -15 dB SPL to 105 dB SPL, with an added 20 dB-wide plateau of high-probability stimulus region (Fig. 2A, filled area). At every 100 ms during the experiment a new sample was drawn from the distribution to set the amplitude of a pure tone, with its frequency matching the characteristic frequency of the studied neuron (primary auditory cortex of marmoset monkey). The dynamic rate-level function was measured and the coding accuracy (the Fisher information) was calculated (Fig. 2A, solid line), see Watkins and Barbour^9^ for more details. The coding accuracy adaptation was determined for four different positions of the plateau, centered at 5, 25, 45 and 65 dB SPL respectively (Fig. 2A–D). The peak coding accuracy does not align with frequently occurring low sound intensities (Fig. 2A).

The same experimental data evaluated on Riesz’s scale yield far better alignment of coding accuracy with stimuli statistics, especially for low intensities (Fig. 2E). On the other hand, the existing match for high levels (Fig. 2C,D) is preserved (Fig. 2G,H) due to the similarity of both scales for high intensities (Fig. 1). The match between the stimulus statistics and the coding accuracy can be quantified by the ratio of the maximal Fisher information in the high-probability region to the global maximum of the Fisher information. For the four examined cases of the plateau centered at (5, 25, 45, 65) dB SPL we obtain the following values of this ratio: (0.46, 0.96, 1, 1) on the pressure level scale, and (1, 1, 1, 1) on Riesz’s scale. Note that the non-uniform shape of the high-probability regions results from the transformation rule for the probability density function.

## Discussion

The described adaptation of neural coding precision to the local stimulus distribution results in a more efficient representation of the environment^5^,^9^. However, the investigation of coding strategy should also take the actual perception intensity into the account^11^. In all likelihood, coding precision expressed by employing the psychophysical scale (such as Riesz’s scale) is more useful and natural than when evaluated in the standard metric system (such as dB SPL). The reasoning is that Riesz’s scale is linear in the true perception intensity as described in the Methods section. Consequently, the smallest noticeable increment in perception Δ*ψ* is proportional to a fixed value on Riesz’s scale, and this value is constant for all stimulus intensities. Hence the unique feature of a stimulus unit based on the psychophysical scale is that the coding accuracy evaluated in such units can be meaningfully compared for different stimuli intensities – unlike the metric scale case. Even if coding precision varies with the stimulus intensity on the metric scale substantially, these variations might be immaterial provided that the actual difference in sensation falls within the smallest noticeable increment.

Note that if Weber’s law was valid for the sound intensity perception, the dB SPL scale would correspond to the exact psychophysical scale. From this point of view the shifted-logarithm in Eq. (7) represents a seemingly negligible correction. We have shown, however, that the difference between Riesz’s and sound pressure level scales affects the coding accuracy adjustment substantially. Ries’z correction, *ψ* ∝ log(*const.* + *I*), to the purely logarithmic Fechner’s law in Eq. (2) has a long history and is more fundamental and general, going beyond the case of the sound intensity perception. See^34^,^35^,^36^ for a detailed account. For example, the equation for Riesz’s scale as a function of the sound pressure in Eq. (7) is formally identical to the psychophysical mel scale^37^, which describes the perception intensity for sound frequency. Both the mel and Riesz’s psychophysical scales thus follow Weber’s law for large stimuli values only.

Our message, however, reaches beyond the topic of psychophysical scales and auditory neuroscience. We argue that the coding accuracy is generally a relative quantity, with respect to chosen units, a fact whose consequences seem to have been neglected in the experimental research. The expected matching of stimulus statistics with the coding accuracy is thus not absolute and does not hold in different unit systems. The coding accuracy reflects the spread of estimated stimulus values, which is affected not only by the stochastic nature of neural responses but also by the arbitrarily chosen unit system for stimulus quantification. In addition, we believe that coding accuracy should generally be evaluated on the scale which is linearly proportional to the internal representation of the stimulus, i.e., proportional to the actual perception intensity.

Finally, it is worth noting that different ways to asses the neural coding efficiency were developed over the decades. A substantial part of the literature employs Shannon’s measure of information^38^ to determine the absolute scale on neuronal performance^39^. By treating the neuronal system as an information channel, and by maximizing the mutual information between stimuli and responses, one obtains the optimal stimulus distribution, as for example in^40^,^41^,^42^,^43^,^44^. Under the assumption of vanishing response variability, the optimal stimulus distribution is proportional to 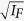
45,46,47,48,49,50,51, which is known to be invariant under coordinate transformations^33^. Heuristically, one may view this result as providing support for the idea of high coding precision matching high probability stimulus regions^40^. Unlike the local method of Fisher information described in this paper, however, the information theory determines the complete (global) form of the stimulus distribution.

## Additional Information

**How to cite this article**: Kostal, L. and Lansky, P. Coding accuracy on the psychophysical scale. *Sci. Rep.* **6**, 23810; doi: 10.1038/srep23810 (2016).
